# Innovations in Gritti-Stokes Amputation: A Focused Analysis of Immobile Patient Outcomes

**DOI:** 10.7759/cureus.69998

**Published:** 2024-09-23

**Authors:** Mahmoud Okaz, Mohamed S M Elshikhawoda, Hisham Abdelrheem Abdelrhman Hamad, Sohaib Jararaa, Abdillahi Ahmed Roble, Ehsan Nawaz, Heledd Catrin Thomas, Muhammad Numan Zahid, Waseem Ahmad, Tarig Barakat

**Affiliations:** 1 Vascular Surgery, Glan Clwyd Hospital, Rhyl, GBR; 2 Surgery, University of Bahri, Khartoum, SDN; 3 Vascular and Endovascular Surgery, Glan Clwyd Hospital, Rhyl, GBR; 4 Colorectal Surgery, Royal London Hospital, London, GBR

**Keywords:** amputation, gritti-stokes amputation, major lower limb amputation, peripheral vascular diseases, through the knee amputation

## Abstract

Background

Major lower limb amputation is a surgical operation performed for different reasons, including life-threatening infection, ischemia, trauma, and tumors. This study aims to investigate the viability of Gritti-Stokes amputation as a suitable alternative for individuals with impaired mobility and peripheral vascular disease.

Patients and methods

This was a descriptive, retrospective, and observational study. All patients who met the criteria and underwent Gritti-Stokes amputation were included. The study had a duration of one year, commencing in December 2022 and concluding in December 2023. The data was processed using statistical software (SPSS, version 21; IBM Corp., Armonk, NY, US).

Results

Twenty-four patients were included. Concerning the complications, 16 patients (66.7%) did not experience any complications, while 7 patients (29.2%) had stump infections. Three of them ultimately required more extensive amputation. A phantom limb was observed in a single patient, accounting for a mere 4.2% of the total. Total of 21 patients, 87.5% reported great stability and lever motion when transitioning from bed to chair, as well as vice versa. Furthermore, wound healing was observed in 21 individuals (87.5%), encompassing both primary and secondary wound healing.

Conclusion

The Gritti-Stokes amputation provides patients with excellent stability and lever function to facilitate movement. The Gritti-Stokes amputation is a suitable option for immobile and high-risk patients.

## Introduction

Peripheral vascular disease is one of the major causes of major lower limb amputations [1]. More than 200 million people are diagnosed with peripheral vascular disease globally [2]. The prevalence of patients with peripheral vascular disease who underwent amputation was 4% [3].

The 2023 national vascular register documented a rise in the number of major amputations, namely, 3,505 major lower limb amputations. However, the percentage of through knee amputations was only 3.7% [4].

The Gritti-Stokes was first presented by Rocco Gritti in Milan in 1857. He described the use of the patella as a weight-bearing surface [5]. In 1962, Martin and Wickham presented the initial findings of a study involving a group of patients. They raised concerns over the appropriateness of the stump for a prosthesis [6]. Following a period of 20 years, Weale et al. and Newcombe et al. agreed with the determination that the Gritti-Stokes amputation was not a viable alternative. On the contrary, they promoted the through-knee technique as a more suitable option [7,8].

The Gritti-Stokes amputation procedure provides patients with a lengthier stump, which serves as an effective lever for wheelchair and bed moves. However, several challenges exist, including prosthetic fitting, patella instability, cosmetic appearance, and the surgeon's conformability and training in performing the procedure.

In this study, we aim to investigate the viability of the Gritti-Stokes amputation as a suitable alternative for individuals with impaired mobility and peripheral vascular disease.

## Materials and methods

Study design and population

A retrospective analysis of the maintained database was performed on all adult patients who underwent a Gritti-Stokes amputation between December 2022 and December 2023 at a vascular surgery department in Glan Clwyd Hospital in North Wales.

Data collection

We retrieved the data of the patients who underwent a Gritti-Stokes amputation at Glan Clwyd Hospital between December 2022 and December 2023.

The clinical evaluation included factors such as the patient's age, gender, comorbidities, smoking, presentation, and outcome.

Inclusion and exclusion criteria

The Gritti-Stokes amputation was offered only for immobile patients such as those with bilateral amputations, paraplegia, patients deemed unsuitable for prostheses, and high-risk patients.

The exclusion criteria were any patient who was planned to have the procedure and end up with higher amputations.

Procedure description

Patient Preparation and Position

The patients were supine.

Flap Marking

The landmarks for the flap were the medial and lateral femoral condyles and the line drawn from this point to the distal of the patella for the longer superior flap and shorter inferior flap toward the popliteal fossa.

Incision and Bone Exposure

This was a U-shaped incision with mobilization of the patella and cutting of the muscles covering the condyles.

Bone Sawing and Vascular Control

After cutting the bone proximal to the femur condyles, the popliteal neurovascular bundle will be exposed and secured by suturing it with absorbable suture material.

Closure of the Stump

We performed an excision of the inferior patellar ligament and filed the lower surface of the patella until all soft cartilaginous tissue was stripped away and then closed the patellar gap.

We then drilled four holes in the patella and the femur and tied the patella to the femur by using loop polydioxanone suture (PDS) 1. Closure of popliteal fossa by muscles and closure of the skin flap subcuticular by using Monocryl 3/0.

Data analysis

The data collected for this study was processed using SPSS 21 software (IBM Corp., Armonk, NY, US), including data entry, cleaning, and analysis. Descriptive statistics were utilized to present the frequency tables with corresponding percentages. The mean and standard deviations were also reported.

## Results

We enrolled a total of 22 patients. The majority of the patients were male, accounting for 79.2% (n = 19), and had a mean age of 76.5 ± 7.8 years SD. All the patients were smokers and had additional medical conditions, including hypertension (66.7%) and ischemic heart disease (66.7%). Nevertheless, the remaining comorbidities were less common (Table 1).

**Table 1:** Patient demographics and comorbidities DM: Diabetes Mellitus; CKD: Chronic Kidney Disease; IHD: Ischemic Heart Disease; COPD: Chronic Obstructive Pulmonary Disease

Variable		Number	Percentage
Gender	Male	19	79.2%
	Female	05	20.8%
Smoking	Yes	24	100%
	No	0	00%
DM	Yes	10	41.7%
	No	14	58.3%
Hypertension	Yes	16	66.7%
	No	8	33.3%
CKD	Yes	3	12.5%
	No	21	87.5%
IHD	Yes	16	66.7%
	No	8	33.3%
COPD	Yes	10	41.7%
	No	14	58.3%
Total		24	100%

The majority of the patients had undergone prior interventions, either for revascularisation and/or amputation. However, the majority of them underwent endovascular intervention (50%) (Table 2).

**Table 2:** Intervention history AKA: Above Knee Amputation

Variable		Number	Percentage
Previous intervention	Yes	16	66.7%
	No	8	33.3%
Option of revascularization			
	Open	4	16.7%
	Endovascular	12	50%
	No intervention	8	33.3%
Previous amputation			
	AKA	06	25%
	No amputation	18	75%
Total		24	100%

Concerning the complications, 16 patients (66.7%) did not experience any complications while 7 patients (29.2%) had stump infections. Three of them ultimately required more extensive amputation and the rest of them were treated with antibiotics and wound dressing. A phantom limb was observed in a solitary patient, accounting for a mere 4.2% of the total. Total of 21 patients, 87.5% reported great stability and lever motion when transitioning from bed to chair, as well as vice versa. Furthermore, wound healing was observed in 21 individuals (87.5%), encompassing both primary and secondary wound healing.

The operative time mean was 47+ 25 months SD. Moreover, the mean length of hospital stay was 14+19 days SD. Till the end of this study, no mortality was reported among the patients.

## Discussion

Nearly one-third of our patients developed stump infection (seven patients) with secondary healing reported in only four of them while the rest of the three patients ended up with higher amputation. The majority of authors who developed stump infection in variable degrees reported healing in 100% of their cases; this discrepancy might be related to the degree of severity of vascular disease [6,7,9].

The study conducted by Houghton et al. revealed that the recovery rate for the Gritti-Stokes amputation was comparatively lower than that of the through knee amputation. This is in contrast to our investigation, which was conducted as part of his evaluation of the prosthesis fitting at that time [10].

Yusuf and his colleagues conducted a study on 144 patients who underwent a Gritti-Stokes amputation. They found that 30% of these patients were able to regain mobility and successfully fit a limb. Additionally, they observed a lower rate of higher amputations. Furthermore, they concluded that there was no significant difference in mortality rates when compared to below-knee amputation. They also suggested that Gritti-Stokes could be a suitable alternative if below-knee amputation is not possible. This is consistent with our study [11].

Lim C et al. conducted an assessment of the Gritti-Stokes amputation in older patients. They found a mortality rate of approximately 25%, successful primary and secondary wound healing, and no instances of phantom limb were reported. Nevertheless, he documented the occurrence of stump infection, hemorrhaging, and necrosis of the skin on the stump. Our investigation supports the notion that the GSA is a viable substitute for older people [12].

A study evaluated the Gritti-Stokes amputation in traumatic patients, reporting limb fitting in up to 92% of the patients, with only one patient experiencing a stump infection and one developing a phantom limb. This study is quite different from ours, as the population consisted of young patients with minimal comorbidities and the absence of peripheral vascular disease [13].

Theriot et al., in their case series involving 15 patients, advocated for the use of Gritti-Stokes in all situations due to its high success rate in healing with few sequelae. However, it is worth noting that only 27% of the patients included in the study had peripheral vascular disease (PVD) [14].

In their study, Brügger et al. examined the five-year survival rate and its correlation with limb fitting. They found that the Gritti-Stokes amputation is highly associated with improved five-year survival, and it is the only component that is linked to limb fitting. The disagreement lies in the fact that one study solely examines the five-year survival rate without considering other parameters, and we did not provide the option of limb fitting to the patient [15].

Our study did not report any stump-related long-term complications such as persistent ulcers, mobile patella, chronic pain, or osteomyelitis. This is not aligned with other studies. However, our study focused on immobile patients, therefore, prothesis-related complications were not reported, and our study was a short-term follow-up study [6,7,9,13,16-17].

The Gritti-Stokes amputation has been criticized, and this might be due to a lack of conformability of the surgeon with it; moreover, the problems with prosthesis fitting were an issue, now with the recent advance in prosthesis developments and the presence of 3D printing technology might lead to a different look for a Gritti-Stokes amputation in the near future [18,19].

Study limitations

The limitations of this work are its retrospective design and the consequent challenges in collecting data. Furthermore, the data gathered for this study was extensive and exclusive to a particular facility, with a limited number of cases. Another constraint of this study is its limited scope, which was deliberately designed to concentrate on immobile patients. Therefore, it is necessary to do a long-term follow-up to report on the outcomes after five years.

## Conclusions

Based on our study, which included reports with a short follow-up period of five years, we have found evidence that a Gritti-Stokes amputation has a low mortality rate and a high wound healing rate.

The Gritti-Stokes amputation provides patients with excellent stability and an effective lever for wheelchair and bed moves. The Gritti-Stokes amputation is a suitable alternative option for immobile and high-risk patients in comparison with other higher amputations. 
